# Root exudate monosaccharides modulate the pathogenicity of *Ralstonia pseudosolanacearum*

**DOI:** 10.3389/fmicb.2025.1662342

**Published:** 2026-01-07

**Authors:** Keli Fang, Dongdong Zhang, Benqiang Zhang, Xikun Li, Yang Zhang

**Affiliations:** Shandong Tobacco Industry Co. Ltd., Jinan, Shandong, China

**Keywords:** *Ralstonia pseudosolanacearum*, pathogenicity, monosaccharides, galactose, transcriptome analysis

## Abstract

The *Ralstonia solanacearum* species complex (RSSC) is a devastating soil-borne pathogen that causes bacterial wilt in solanaceous crops. However, the response of RSSC to monosaccharides—key components of root exudates released into the rhizosphere—remains unclear. We supplemented culture media with several typical monosaccharides derived from tobacco root exudates to mimic rhizosphere signals and examined their effect on the expression of bacterial virulence factors. Transcriptome analysis revealed that galactose markedly altered the physiological status of RSSC, inducing a state resembling that during plant infection. Among the monosaccharides tested, galactose specifically up-regulated the expression of type III secreted effectors. Notably, galactose also actively induced nitrogen metabolism, leading to the production of nitrous oxide and other reactive nitrogen species, which may, like reactive oxygen species, enhance pathogenicity. Furthermore, metabolites extracted from galactose-induced RSSC cultures triggered cell death when infiltrated into tobacco leaves.

## 1 Introduction

Bacterial wilt, caused by plant pathogenic *Ralstonia solanacearum* species complex (RSSC), is one of the most important diseases affecting the production of many important crops worldwide (Abdurahman et al., 2019; Jiang et al., 2017; Okiro et al., 2022; Ray et al., 2024; Sharma et al., 2021; Suhaimi et al., 2017; Thano and Akarapisan, 2018), particularly economically important crops tobacco, tomatoes, potatoes, and bananas. RSSC normally infiltrates plant roots from the surface or soil via wounds or natural openings from which secondary roots emerge, and colonizes the xylem vessels and then blocks plant water flow, leading to wilting symptoms (Genin, 2010; Hikichi et al., 2017; Vasse et al., 2000). After invasion, RSSC cells attach to the exteriors of host plant cells and inject large repertoires of type III effectors (T3Es) into plant cells (Hikichi et al., 2017). RSSC can persist in soil for extended periods until activated by host-derived signals. Chemotaxis—the directed movement of motile bacteria in response to— chemical agent, is a common phenomenon (Zhou et al., 2021). Bacteria can detect and respond to a wide range of chemical stimuli, including amino acids, carbohydrates, organic acids, aromatic chemicals, and phosphate (Colin et al., 2021). For plant pathogens, recognition of the host plant via root exudates is the initial step for successful invasion. Bacterial chemotaxis is also a key precursor to ecological interactions such as symbiosis, infection, and root colonization (Hasegawa et al., 2019).

Root exudates comprise a complex mixture of organic and inorganic substances released into the rhizosphere (Bais et al., 2006). Plants allocate up to 40% of their photosynthates to the rhizosphere, including low-molecular-weight compounds such as amino acids, organic acids, sugars, and other secondary metabolites, as well as high-molecular-weight compounds like polysaccharides and proteins (Bais et al., 2006). For instance, L-glutamic acid from host plants has been proven to be the key active component, which promotes colonization of *R. solanacearum* cells in the roots and stems and accelerates disease incidence of tomato plants (Shen et al., 2020). Galacturonic acid released from plant cell walls can be used to nourish bacterial pathogen cells and accelerate the disease progression of bacterial wilt (González and Allen, 2003; Huang and Allen, 1997; Huang and Allen, 2000). Tryptophan and methionine are utilized by *R. solanacearum* to enhance virulence (Brown and Allen, 2004; Genin and Boucher, 2004; Plener et al., 2012). In addition, *organic acids such as cinnamic, myristic, and fumaric acids from root exudates induce biofilm formation and modulate bacterial motility* (Li et al., 2017). Fructose, mannose, and arabinose induce biofilm formation of strain OE1-1 in the minimal 1/4 M63 media (Mori et al., 2018a; Mori et al., 2018b). Root exudates can also activate the syrB gene to synthesize the phytotoxin, syringomycin, by *Pseudomonas syringae* pv syringae (Mo et al., 1995). Root exudates are sensed by methyl-accepting chemotaxis proteins (MCPs), which are transmembrane chemoreceptors on the bacterial cell surface (Hida et al., 2015; Tunchai et al., 2021). Upon ligand binding, MCPs initiate a signaling cascade through chemotaxis (Che) proteins to regulate flagellar motility. Genomic analyses indicate that RSSC strains encode over 20 MCPs. For example, strain Ps29 expresses 22 MCPs, two of which function as aerotaxis sensors (Hida et al., 2015), while three others—McpA, McpM, and McpT—mediate chemotaxis toward amino acids, L-malic acid, and D-malic acid, respectively (Hida et al., 2015; Tunchai et al., 2017). McpM-mediated chemotaxis has been shown to be essential for full virulence in tomato (Hida et al., 2015; Tunchai et al., 2017). Additionally, the RS_RS07350 homolog (designated McpC) and McpP have been identified as citrate chemoreceptors in R. pseudosolanacearum Ps29, with the latter also mediating positive chemotaxis to phosphate and negative chemotaxis to maleate (Hida et al., 2019).

Gene expression analysis has played a critical role in determining the various virulence factors in *R. solanacearum* (de Pedro-Jové et al., 2021). Monosaccharides (D-glucose, D-mannose, D-fructose, and D-galactose) have been proven to activate the production of ralfuranones in strain OE1-1. In order to investigate the effect of monosaccharides released from the root on the expression pattern of the major pathogenic factors of *R. pseudosolanacearum* WXQ_10, we selected six monosaccharides to culture the cells and perform transcriptome profiling of *R. solanacearum*. Comparative transcriptome analysis revealed that the differentially expressed genes of the strain WXQ_10 cultured in galactose are mostly enriched in the virulence factors, for example, the type III secretion system, type III effectors. We further investigated the roles of nitrogen metabolism, for example, denitrification, suggesting the reactive nitrogen species, like the reactive oxygen species, might be the key factors to cause the pathogen occurrence. This study underscores the importance of redox balance in the process of virulence cultured in galactose. Beyond the cell-to-cell interactions, the metabolites produced by RSSC under the addition of different monosaccharides also demonstrated different pathological characteristics by injected into plant leaves.

## 2 Materials and methods

### 2.1 Bacterial culture and growth conditions

*Ralstonia pseudosolanacearum* strain was cultured in nutrient agar media (beef extract 3.0 g, yeast extract 1.0 g, peptone 5.0 g, glucose 10.0 g, agar 20 g, pH 6.8–7.0 in 1000 ml ddH_2_O) in Petri plates and incubated for 3 days at 28 °C in an incubator. After 3 days, the creamy white colonies of *R. pseudosolanacearum* were taken, dissolved, and incubated in MB media. Modified B medium (pH 7.0) was prepared by dissolving peptone (10.0 g), yeast extract (1.0 g), and tryptone (1.0 g) in 1000 ml of water. This is prepared for cultivation and the draft genome sequencing of WXQ_10 by Shanghai Personalbio Technology Co., Ltd.

MGRL media contains 1.75 mM sodium phosphate buffer (pH 5.8), 1.5 mM MgSO_4_, 2.0 mM Ca(NO_3_)_2_, 3.0 mM KNO_3_, 67 μM Na_2_EDTA, 8.6 μM FeSO_4_, 10.3 μM MnSO_4_, 30 μM H_3_BO_3_, 1.0 μM ZnSO_4_, 24 nM (NH4)_6_Mo_7_O_24_, 130 nM CoCl_2_,1 nM CuSO_4_. MGRLS media were prepared by supplementation of 3% sucrose (Kai et al., 2014).

For RNA extraction, cells were first cultured in liquid MB media to an OD600 nm of 2.0, then centrifuged, and the supernatant was discarded. The precipitate was dissolved in MGRL media and inoculated into MGRLS media with monosaccharide addition, final OD600 nm was approximately 1.0. The cell solution is cultured in MGRLS media with 0.5% mannose, 0.5% L-arabinose, 0.5% xylose, 0.5% fructose, 0.5% glucose, and 0.5% galactose (final concentration) in a flask, respectively, then incubated in a rotary shaker at 180 rpm speed, 28 °C temperature for 4 h to prepare for the RNA sequencing. The supernatant was discarded, and the pellet was frozen in liquid nitrogen and stored at −80 °C for RNA extraction by Majorbio Bio-pharm Technology Co., Ltd. (Shanghai, China).

### 2.2 Pathogenicity on tomato and chili plants

*Ralstonia pseudosolanacearum* was cultured in LB medium overnight at 28 °C in a rotary shaker at 180 rpm. The cells were harvested by centrifugation at 6000 rpm and 4 °C in a centrifuge machine. The cells were dissolved in 10 ml of distilled water inside Eppendorf tubes using a vortex mixer, and the resultant cell solution was diluted in water. The OD600 nm was adjusted to 0.8. For inoculation, a bacterial suspension (10^9^ CFU·ml^−1^) was applied at 10 ml per pot. Four equidistant holes were first made in the soil using a 1 ml pipette tip, and the suspension was poured evenly into these holes. The inoculated plants were placed at 30 °C in a growth chamber for the development of characteristic disease symptoms.

### 2.3 Draft genome sequencing, RNA sequencing, and data analysis

The genomes of strain WXQ_10 were sequenced using the Illumina NovaSeq platform. Libraries with a fragment length of 150 bp were constructed using TruSeqTM DNA Sample Prep Kit. High-quality reads were used for *de novo* genome assembly with A5-MiSeq (Coil et al., 2015) and SPAdes (Bankevich et al., 2012) to construct contigs and scaffolds, respectively. tRNA and rRNA and non-coding RNA were predicted by software tRNAscan-SE v1.3.1,^1^ Barrnap v0.9 rRNA,^2^ and obtained from the comparison to Rfam database (Kalvari et al., 2018). The draft whole genome sequence was deposited in NCBI (BioProject PRJNA796546) accession number JAKKIF000000000.

Original sequencing data of Illumina HiSeq contained some data of low quality. After removing the adapter sequence, trimming the ends of reads with low sequencing quality, removing reads containing 10% N, and discarding the small fragments with a length less than 25 bp after adapter and mass pruning, the clean data were used for genome assembly. Clean reads were compared with Rfam database to measure the pollution of ribosomal RNA. Software RSEM^3^ was used to quantify the expression of a gene or RNA. The data were analyzed on the online platform of Majorbio Cloud Platform,^4^ Shanghai Majorbio Bio-pharm Technology Co., Ltd. A gene was considered differentially expressed in the two conditions if its expression level was >2-fold different with an adjusted *p* value of <0.05. Based on the sequence information, gene functional annotation, differential expression analysis, GO, and KEGG pathway enrichment analyses were performed on the platform described above. The raw data of transcriptome were deposited in NCBI (The BioProject accession number is PRJNA798162, accession number SRR17649157-SRR17649163).

### 2.4 RNA extraction and cDNA synthesis from WXQ_10 cultured in monosaccharides

TransZol Up RNA kit (ET111-01) by TransGen Biotech Co., Ltd. (Beijing, China) was used for the extraction of RNA from WXQ_10 strain. For qPCR analysis, samples were collected at 1, 4, and 12 h after inoculating bacteria (initial OD₆₀₀ = 1.0) into MGRLS media with monosaccharides. The sample collected at the 4-h time point was additionally used for transcriptome analysis, and 1.8 ml of the sample was centrifuged at 5000 rpm at 4 °C for 10 min. After removing the supernatant, the pellet was frozen in liquid nitrogen and stored at −80 °C for RNA extraction. Three replications of each sample were used for RNA extraction. RNA concentration and purity were determined via microspectrophotometry (NanoDrop, Thermo Fisher Scientific, Wilmington, DE).

TransScript All-in-One First-Strand cDNA Synthesis SuperMix for qPCR AT341-02 (One-Step gDNA Removal) was used for cDNA synthesis from TransGen Biotech Co., Ltd. For cDNA synthesis, 4 μl of extracted RNA was mixed with 10 μl of RNase-free water and incubated in a water bath at 65 °C for 5 min. Then added 1 μl of genomic DNA remover and 4 μl of 5X transcript All-in-one super mix for qPCR. Incubate the mixture at 42 °C for 15 min, then again incubate it at 85 °C for 5 s to inactivate enzymes.

Absolute quantification using quantitative PCR (qPCR) reactions was performed in duplicate 25 μl reactions with PerfectStart® Green qPCR SuperMix TG-AQ601 (TransGen Biotech Co., Ltd., Beijing, China) and included 1 × master mix, 400 nM forward and reverse primers for every amplification fragment, and 50 ng template cDNA. Reactions were carried out on an ABI PRISM 7300 real-time PCR system (Applied Biosystems) with reaction parameters of 10-min polymerase activation, followed by 40 cycles, with 1 cycle consisting of 15 s at 95 °C and 1 min at 57 °C. Standard curves were made for absolute quantification using 10-fold dilutions (2.5 to 0.0025 μg cDNA) from RNA collected and then reverse transcribed from WXQ_10 cells with an OD600 value of 1.0 and on MGRLS media with monosaccharide for the expression of the genes. The results of qPCR were shown in average ± SD at three different time points.

### 2.5 Assessment of metabolite effects on tobacco and tomato leaves

*Ralstonia solanacearum* strains were cultured in MGRLS medium (300 ml flasks) at 28 °C and 180 rpm for 4 days. Bacterial metabolites were extracted three times with equal volumes of ethyl acetate, and the combined extracts were dried under rotary evaporation. The residues were dissolved in 500 μl methanol for tobacco inoculation or in 50 μl methanol plus 450 μl 0.9% NaCl for detached tomato leaves, respectively. Tobacco, tomato, and chili plants were grown under controlled conditions (25 °C, 16-h light/8-h dark, 10,000 lux). Three- to four-week-old plants were inoculated by injecting 50 μl of metabolites into the third leaf axil after creating two micro-wounds with a sterile syringe; alternatively, detached tomato leaves were incubated in Petri dishes for 72 h with petioles submerged in the test solution to check the plant virulence of the extracts. All inoculated materials were maintained in an incubator for 3 days.

## 3 Results

### 3.1 Verification of plant disease on tomato and genome sequencing of WXQ_10

**Figure 1:**
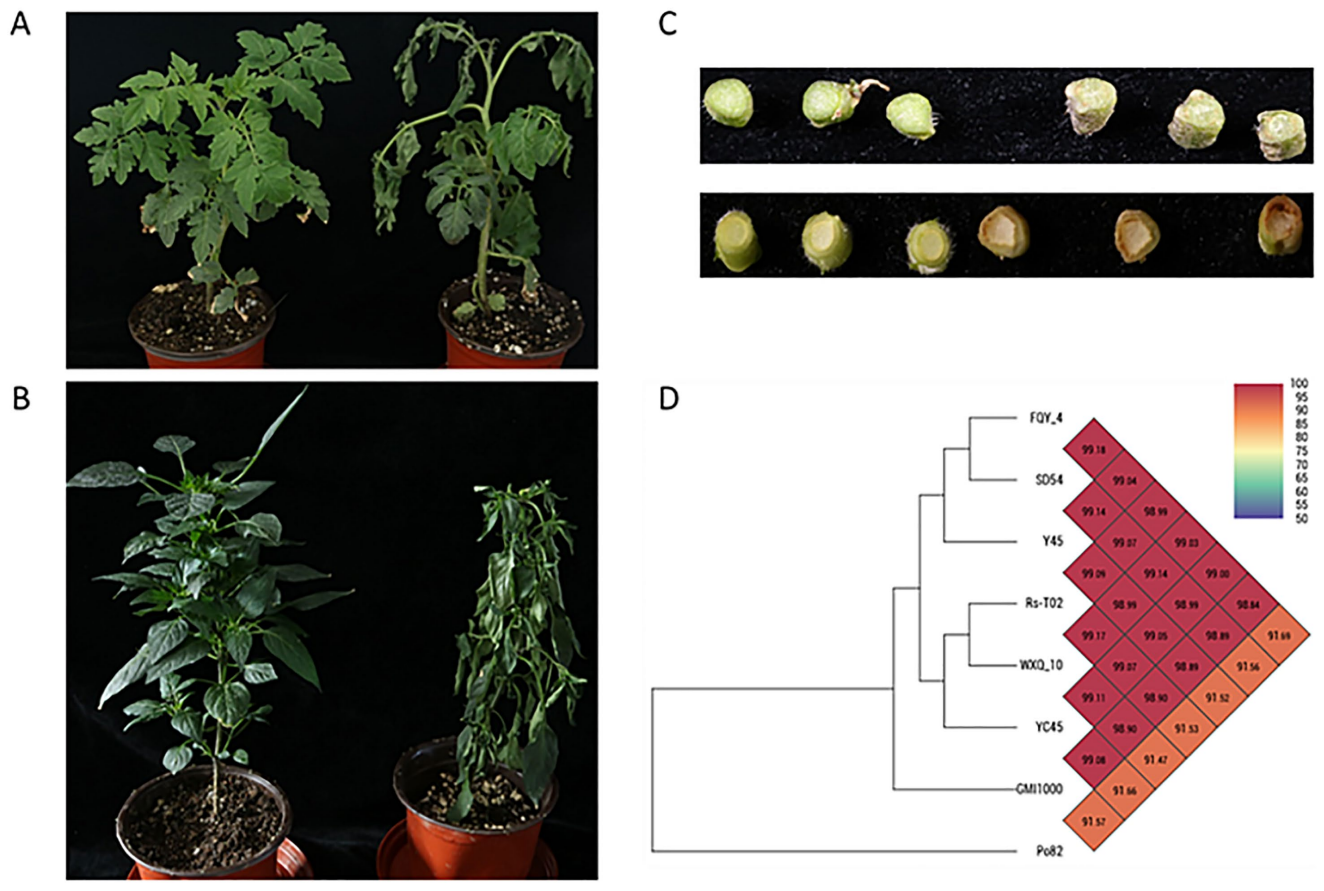
Verification of plant disease on other solanaceae crops and a phylogenetic tree prepared using genomes of WXQ_10 and 7 reference RSSC strains based on OrthoANI values. **(A,B)** Wilting symptoms appeared from the top of plant; **(C)** Stem infection (brown vessels) caused by WXQ_10, the control of transverse section of three stems was shown in left, the infected in right; **(D)** phylogenetic tree: strains and their accession number shown in the brackets included *Ralstonia solanacearum* GMI1000 (AL646052), *Ralstonia solanacearum* Rs-T02 (LKVH00000000), *Ralstonia solanacearum* YC45 (CP011997 and CP011998), *Ralstonia solanacearum* FQY_4 (CP004012 and CP004013), *Ralstonia solanacearum* SD54 (ASQR02000000), *Ralstonia solanacearum* strain Po82 (CP002819 and CP002820), *Ralstonia solanacearum* Y45 (AFWL00000000).

In order to prove that WXQ_10 can also cause diseases in other solanaceae crops, we watered the seedlings of tomato and chili plants via root infection. The plants subsequently developed wilting symptoms beginning from the top leaves (Figure 1A), confirming the virulence of WXQ_10. To determine its taxonomic position, we sequenced the whole genome of WXQ_10, generating a total of 1,098 Mbp of paired-end (150 bp) reads. Assembly and annotation metrics are presented in Supplementary Table S1. The complete genome is 5,638,120 bp in length with a GC content of 67.05%, and encodes 4,973 predicted coding sequences. In addition, 51 tRNAs, three rRNAs (5S, 16S, and 23S), and 82 non-coding RNAs were identified (Supplementary Table S2).

The *Ralstonia solanacearum* species complex (RSSC) formerly consisted of four phylotypes, and the taxonomic status cannot be reliably resolved using only 16S rRNA sequences. To understand the genetic relationship with the well-studied strains RSSC, we performed genome-wide average nucleotide identity (ANI) analysis. Phylogenetic reconstruction based on ANI values revealed that WXQ_10 belongs to Ralstonia pseudosolanacearum (Figure 1B). According to established criteria, strains with ANI values above 95–96% are considered conspecific. Among the eight strains analyzed, seven—including GMI1000, Rs-T02, YC45, FQY_4, SD54, Y45, and WXQ_10—formed a coherent cluster with pairwise ANI values exceeding 98.9%, supporting their classification as a single species. Notably, WXQ_10 showed 96.77% ANI similarity to the type genome of *R. pseudosolanacearum* (strain UQRS 461 = LMG 9673 = NCPPB 1029), with 83.4% genome coverage. This result further confirms that the widely studied strain GMI1000 should also belong to *R. pseudosolanacearum* (Safni et al., 2014).

### 3.2 Overview of transcriptome analysis of WXQ_10 cultured in monosaccharides

#### 3.2.1 Sequencing data quality control and expression difference analysis

Across the six monosaccharide treatments and the sucrose control, the average raw sequencing data per sample comprised approximately 33 million reads, representing about 5,122 Mbp of raw bases. After adapter removal and quality filtering, an average of 32 million clean reads and 4,005 Mbp of clean bases were retained per sample. The clean data exhibited an average error rate of 0.013%, with Q20 and Q30 values averaging 98.2 and 94.48%, respectively (Supplementary Table S3). The average rRNA content across all seven samples, as determined by Rfam alignment, was 11.19%, with all samples falling below the 15% threshold.

Venn analysis (Supplementary Figure S1) displayed the number of mRNA and sRNA transcripts with expression levels ≥1 TPM (Transcripts Per Million) in each sample. On average, 629 sRNAs, 4,822 mRNAs, and a combined total of 5,451 transcripts were detected per sample. The number of differentially expressed genes (DEGs) for both mRNA and sRNA is also summarized in Supplementary Figure S1. Among the 4,973 protein-coding genes annotated in the WXQ_10 genome, approximately 97.0% were expressed under the experimental conditions tested.

#### 3.2.2 The expression pattern of six monosaccharides compared with the sucrose control

Compared to the sucrose, galactose elicited 487 up- and 546 down-regulated DEGs; L-arabinose induced 334 up- and 176 down-regulated DEGs; mannose resulted in 103 up- and 157 down-regulated DEGs; and xylose led to 268 up- and 217 down-regulated DEGs. In total, 1,689 DEGs were identified across all comparisons. Notably, fructose and glucose treatments each induced fewer than 100 DEGs in both up- and down-regulated categories. In contrast, galactose triggered the most substantial transcriptional changes, with over 500 DEGs in each direction. With respect to sRNA-specific DEGs, the number of up-regulated DEGs in galactose (162) was highest, and galactose again exhibited the strongest effect, showing 162 up-regulated sRNAs—more than twice the number of the second-highest treatment (L-arabinose, 70 up-regulated sRNAs) among a total of 318 sRNA DEGs detected. The number of down-regulated sRNA DEGs in galactose was 45, ranking third among all treatments. Of all the 4,973 coding sequences, 1,326 DEGs were identified in the six samples compared in the sucrose sample.

**Figure 2:**
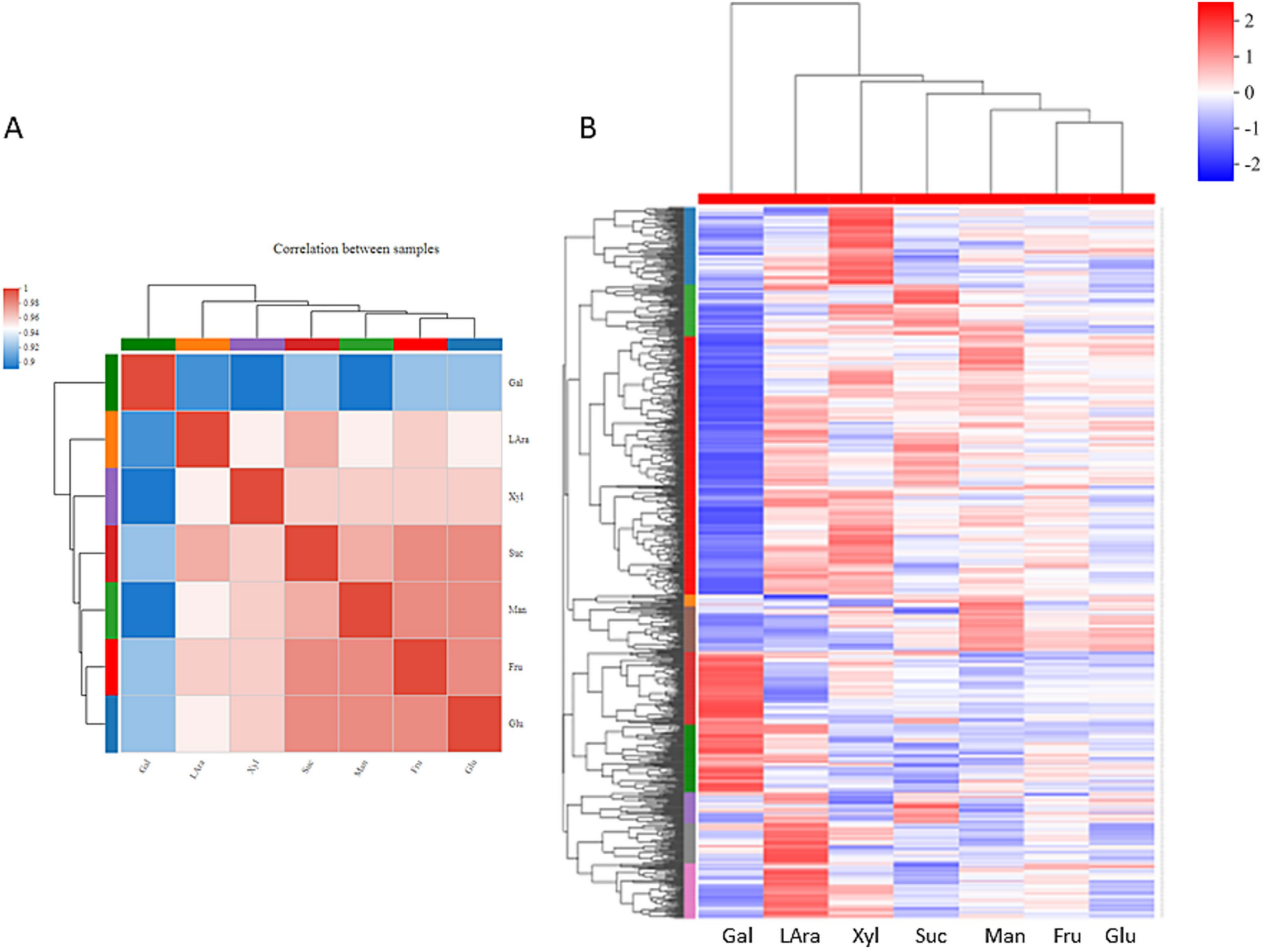
Correlation between samples and heatmap of cluster analysis. **(A)** Correlation between samples; **(B)** each column in the figure represents a sample, and each row represents a gene. The color depth in the heat map represents the expression amount of the gene in the sample. The specific change trend of the expression amount was shown in the number label under the color bar at the upper right. On the left is the tree view of gene clustering. The upper part is the tree view of sample clustering. The color of the color block indicates different groups, and the lower part is the name of the sample. The closer the branches of the two samples are, the closer the expression patterns of all genes in the two samples are, that is, the closer the change trend of gene expression is.

Different from the high expression pattern of sRNA in galactose treatment, we found the number of down-regulated DEGs of mRNA was significantly higher than the others, approximately three times that of the second highest (153 in xylose sample) in down-regulated samples. Compared to the sucrose, the number of up-regulated DEGs was 298, 283, and 242 in galactose, L-Arabinose, and xylose treatments, respectively. The comparison between the sample volcano plot and the sucrose sample is shown in Supplementary Figure S2. Based on the correlation analysis and PCA analysis, we found that the expression pattern of the galactose treatment was different from the other five treatments, compared to the sucrose control, less than 0.94 (Figure 2A). PCA analysis also showed that galactose treatment was greatly different from other treatments (Supplementary Figure S3). Based on the heat map, the galactose treatment has an obvious difference from other treatments compared to the sucrose treatment (Figure 2B).

#### 3.2.3 KEGG enrichment analysis

**Figure 3:**
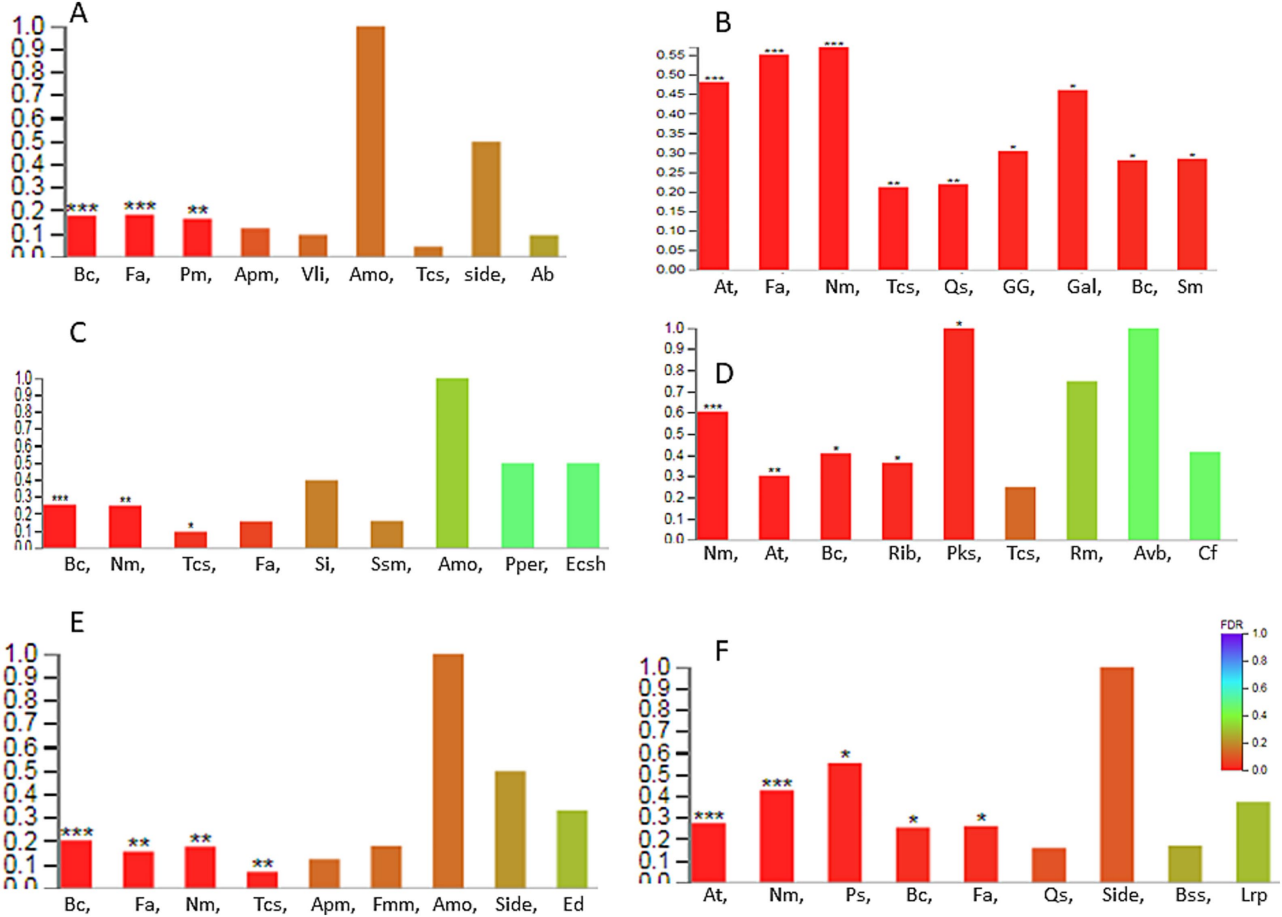
KEGG enrichment analysis. **(A)** Fructose; **(B)** L-Arabinose; **(C)** Mannose; **(D)** Galactose; **(E)** Glucose; **(F)** Xylose. The abscissa represents the name of the path; the ordinate represents the enrichment rate [the ratio of the number of genes annotated to the pathway in the gene set to the number of background genes annotated by all genes. The larger the rich factor, the greater the enrichment degree]. Color indicates the significance of enrichment, i.e., *p*-value. The darker the color, the more significant the enrichment of this pathway is. The mark of *p*-value < 0.001 is ***, the mark of *p*-value < 0.01 is **, the mark of *p*-value < 0.05 is *, and the color gradient on the right indicates the size of *p*-value. Ab: Arginine biosynthesis, Amo: Amoebiasis, Apm: Arginine and proline metabolism, At: ABC transporters, Avb: Acarbose and validamycin biosynthesis, Bc: Bacterial chemotaxis, Bss: Bacterial secretion system, *Cf*: Carbon fixation in photosynthetic organisms, Ecsh: Epithelial cell signaling in *Helicobacter pylori* infection, Ed: Ethylbenzone degradation, Fa: Flagellar assembly, Fmm: Fructose and mannose metabolism, Gal: Galactose metabolism, GG: Glycolysis / Gluconeogenesis, Lrp: Longevity regulating pathway—worm, Nm: Nitrogen metabolism, Pm: Phenylalanine metabolism, Pks: Polyketide sugar unit biosynthesis, Pper: Protein processing in endoplasmic reticulum, Ps: Photosynthesis, Qs: Quorum sensing, Vli: Valine, leucine and isoleucine metabolism, Rib: Ribosome, Rm: Retinol metabolism, Si: *Salmonella* infection, Side: Biosynthesis of siderophore group nonribosomal peptides, Sm: Sulfur metabolism, Ssm: Starch and sucrose metabolism, Tcs: Two-component system.

**Figure 4:**
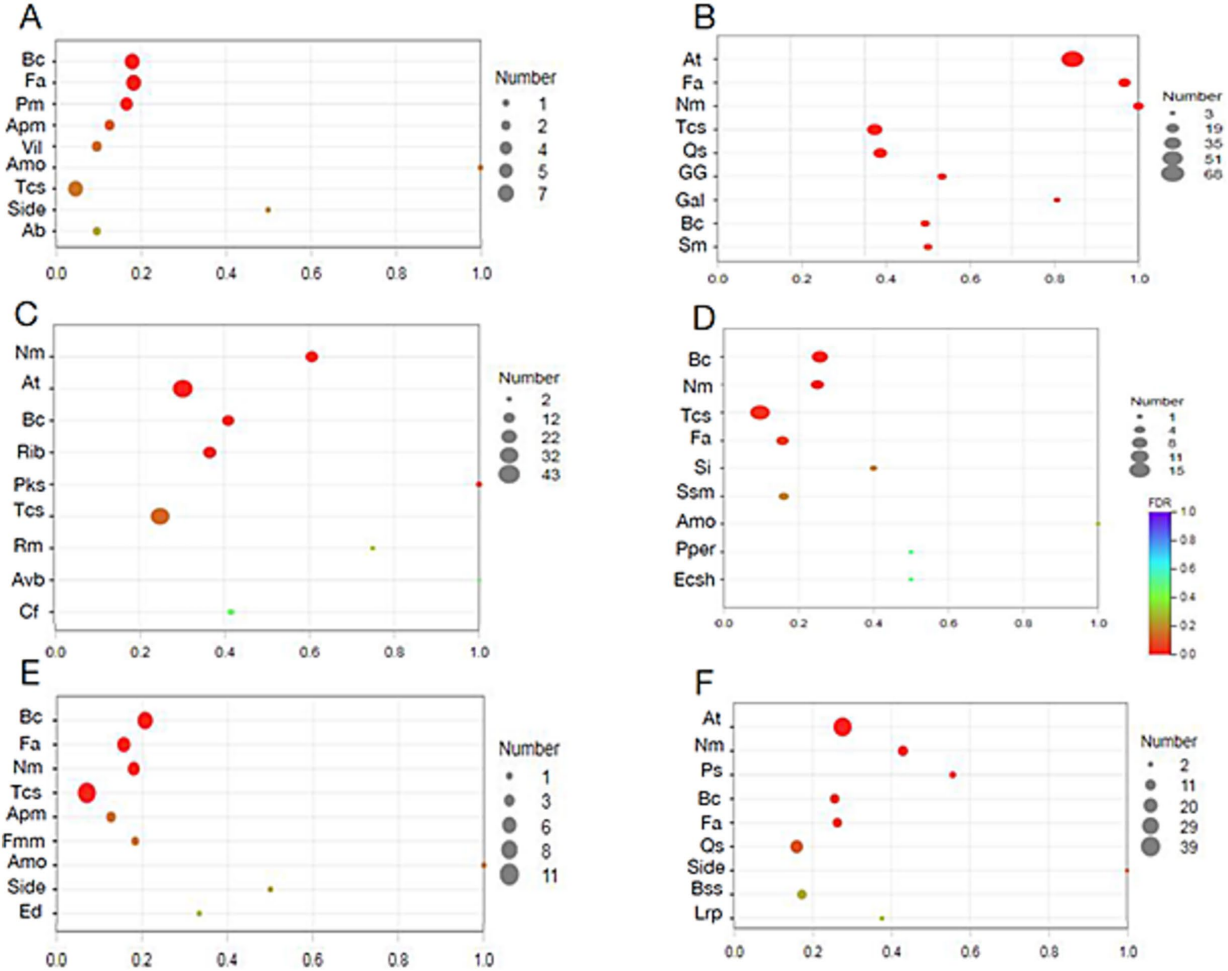
KEGG enrichment analysis. **(A)** Fructose; **(B)** L-Arabinose; **(C)** Galactose; **(D)** Mannose; **(E)** Glucose; **(F)** Xylose. The vertical axis represents the name of the path, the horizontal axis represents the rich factor, the size of the point represents the number of genes in the path in the gene set, and the color of the point corresponds to different Q-value ranges. The abbreviation of the pathway name was same as those in Figure 3.

Compared to the sucrose treatment, the significantly enriched KEGG pathway in fructose, glucose, and mannose samples was less than 0.25 (Figure 3). Specifically, fructose treatment led to significant enrichment in only three pathways: bacterial chemotaxis, flagellar assembly, and phenylalanine metabolism. Glucose and mannose treatments resulted in four pathways (bacterial chemotaxis, flagellar assembly, nitrogen metabolism, and two-component system) and three pathways (bacterial chemotaxis, nitrogen metabolism, and two-component system), respectively. Among these, bacterial chemotaxis was the most significantly regulated pathway (*p* < 0.001) across all three treatments, though its low rich factor (< 0.25) suggests that the corresponding substrate may be sensed by only one methyl-accepting chemotaxis protein (MCP). In contrast, galactose treatment elicited a markedly stronger response, with the rich factor of the top-ranked nitrogen metabolism pathway reaching 0.6 (Figure 3D). Five pathways were significantly enriched, including nitrogen metabolism, ABC transporter, bacterial chemotaxis, ribosome, and polyketide sugar unit biosynthesis (*p* < 0.001). Similarly, xylose treatment also enriched five pathways—ABC transporters, nitrogen metabolism, photosynthesis, bacterial chemotaxis, and flagellar assembly—with nitrogen metabolism and ABC transporters being the most significant (*p* < 0.001; Figure 3F). Additionally, several bacterial secretion systems (types I, II, and VI) were enriched in xylose, though not significantly. Notably, L-arabinose induced the broadest transcriptional reprogramming, with all visualized pathways being significantly enriched (Figure 4B), particularly nitrogen metabolism, ABC transporters, and flagellar assembly (*p* < 0.001). In galactose, xylose, and arabinose treatments, nearly forty genes were enriched in certain pathways, indicating that these three monosaccharides substantially alter the metabolic profile of WXQ_10 upon addition to the culture medium.

### 3.3 DEGs related to the degradation of pectin and lignin, production of reactive nitrogen species, and secretion of type III effectors were significantly up-regulated under the induction of galactose

#### 3.3.1 DEGs involved in the pectin and lignin degradation were up-regulated under the induction of galactose

Plant cell walls are heterogeneous structures, with cellulose microfibrils embedded in a matrix of pectin, hemicellulose, and lignin. To overcome the barrier of the plant cell wall, phytopathogenic bacteria can produce enzymes, which focus on the deconstruction of cellulose, xylan, and pectin, that are capable of degrading cell wall polymers [cell wall–degrading enzymes (CWDEs)]. In WXQ_10, there were 33 glycosyl transferases (GT), 3 polysaccharide lyases (PL), 30 carbohydrate esterases (CE), 17 auxiliary activities (AA), 11 carbohydrate-binding modules (CBM), 44 glycoside hydrolases (GH) identified and annotated by cazy database. Among the total 138 genes encoding active enzymes for carbohydrates, 44 of them were significantly regulated in at least one sample (Supplementary Table S4). In xylose treatment, four of five DEGs were up-regulated (GH28, CE1, CBM32, GH109), and one was down-regulated (GT104). In fructose treatment, only two DEGs were up-regulated (GH28, CE10). One of two DEGs belonging to the CE10 genes in the glucose was up-regulated, the other one was down-regulated. In arabinose, 13 DEGs were identified, 11 DEGs were up-regulated, and only 2 were down-regulated. In mannose, seven of eight DEGs were up-regulated, and only one DEG was down-regulated.

Different from the above samples, which up-regulated DEGs, 18 of the total 27 DEGs were down-regulated DEGs in galactose samples, nine DEGs were up-regulated and belonged to AA1, PL3, PL6, GH23, GH109, CE3, CE4, GT20 family. Most importantly is that DEGs related to pectin digestion and lignin digestion were highest expressed (PL6 gene 1736, log2FC 3.07; AA1_3 gene 3,914, log2FC 2.42) in the galactose sample (Supplementary Table S4).

#### 3.3.2 Nitrogen metabolism was significantly regulated under the induction of galactose

Nitric oxide (NO) and reactive nitrogen species have emerged as crucial signaling and regulatory molecules across all organisms (Jedelská et al., 2021). Nitrogen metabolism was the most significantly enriched in galactose, xylose, and L-Arabinose (*p* < 0.001). Further analysis revealed that the expression pattern of nitrogen recycle genes in galactose was different from that in arabinose. DEGs in galactose from nitrite to the N_2_ pathway are significantly up-regulated. However, that pathway in arabinose was significantly down-regulated (Table 1).

**Table 1:** Log2FC of genes related with nitrogen metabolism.

Gene	Gene name	Gal	LAra	Xyl	Man	Fru	Glu
1,241	narK	5.03	−2.5	2.63	no	no	no
1,242	narK	4.09	−3.01	1.64	no	no	no
1,243	narG	4.53	−2.45	1.38	no	no	no
1,244	narH	3.72	−2.44	no	no	no	no
1,245	narJ	3.17	−2.33	no	no	no	no
1,246	narI	3.04	−1.97	no	no	no	no
1,247	mobB	3.24	−1.6	no	no	no	no
1,248	narX	2.07	no	no	no	no	no
1,249	narL	1.39	−1.07	no	no	no	no
2,446	narK	−2.04	−1.32	−4.13	1.5	no	1.13
2,447	nirB	−1.87	−2.22	−2.92	no	no	no
2,448	nirD	−1.89	−2.58	−1.85	no	no	no
2,449	nirB	−1.78	−2.28	−1.28	no	no	no
2,450	nasA	−1.49	−2.19	no	no	no	no
3,914	nirK	2.42	no	no	no	no	no
3,915		1.92	no	no	−1.25	no	no
3,916	norB	1.78	−1.01	no	no	no	no
1738	nosZ	3.04	−2.34	no	−1.28	no	no
4,427	ncd2	1.55	−1.89	no	−1.14	no	no

no: not significantly regulated.

Under anaerobic or anoxic conditions, bacteria can use nitrate (NO_3_^−^) as the final electron acceptor of the electron transport chain to complete material and energy exchange. Unexpectedly, in aerobic conditions, genes associated with denitrification and anaerobic ammonium oxidation were also actively expressed, especially in galactose treatment (Table 1; Figure 4). Dissimilatory nitrate reduction from nitrite to ammonia was significantly down-regulated.

We found regA and regB, which are related to redox signal, were also up-regulated in galactose treatment, which exists in the regulatory pathway of oxidative phosphorylation. For example, cytochrome c oxidase cbb3-type genes 277–280 and cytochrome bd complex genes 2,703–2,702. This might be the consequence of the production of reactive oxygen species and reactive nitrogen species in the intracellular and extracellular environment.

#### 3.3.3 Type III effectors were mostly up-regulated in the galactose sample

Type III effector proteins were the key factors that caused the plant wilt. We found there are 54 type III effector proteins encoded in the genome of WXQ_10 based on the research of the Non-Redundant Protein Database. However, compared to sucrose, only 29 effectors were differentially expressed in these monosaccharide treatments (Table 2). Among these DEGs, only one gene in galactose media was significantly down-regulated (Table 2). The most obvious phenomenon is that 19 of the total 29 DEGs were significantly up-regulated only in galactose, suggesting that galactose can significantly alter the metabolic pathway of cells in liquid media toward the infected plant.

**Table 2:** Log_2_FC of DEGs of type III effectors.

Gene	Hit_name	Xyl	Gal	Man	Fru	Glu	LAra
473	WP_064477782.1	no	1.46	no	no	no	no
472	WP_071092182.1	no	1.77	no	no	no	no
498	CUV53507.1	no	1.03	no	no	no	no
538	CAD15502.2	no	1.07	no	no	no	1.3
885	WP_011004179.1	no	1.86	no	no	no	no
888	WP_028861698.1	no	2.21	no	no	no	no
890	WP_011004174.1	no	2.38	no	no	no	no
892	BAH04996.1	no	1.93	no	no	no	no
902	WP_011004162.1	no	2.25	no	no	no	no
920	WP_071507263.1	no	1.05	no	no	no	1.12
922	BAH04966.1	no	1.98	no	no	no	1.69
946	WP_028861671.1	no	2.28	no	no	no	no
1,193	WP_087452903.1	no	1.38	no	no	no	no
1,289	AKZ28928.1	no	1.94	no	1.36	1.03	no
1,300	CUV54731.1	no	1.57	no	no	no	no
1,574	WP_011004043.1	1.02	−1.45	no	no	no	no
1732	WP_087452504.1	no	2.21	no	no	no	no
1881	WP_011002803.1	no	1.42	no	no	no	no
1921	WP_011002842.1	4.93	no	no	no	no	2.19
2033	WP_058907288.1	1	no	no	no	no	no
2,163	WP_058907626.1	no	1.13	no	no	no	no
2,451	WP_071623986.1	no	no	no	no	no	1.78
2,454	CUV30333.1	no	1.25	no	no	no	no
2,625	CUV46768.1	no	1.93	no	no	no	no
2,663	WP_043898127.1	1.34	no	no	no	no	1.35
3,447	WP_011003157.1	no	2.01	no	1.06	no	1.11
4,234	CUV47980.1	no	2.31	no	no	no	no
4,392	WP_051048318.1	no	1.81	no	no	no	no
4,830	WP_028854792.1	no	2.19	no	no	no	no

no: not significantly regulated.

### 3.4 Some virulent factors were not significantly up-regulated by the monosaccharides

#### 3.4.1 EPS biosynthetic genes and biofilm biosynthetic genes were not significantly up-regulated

**Figure 5:**
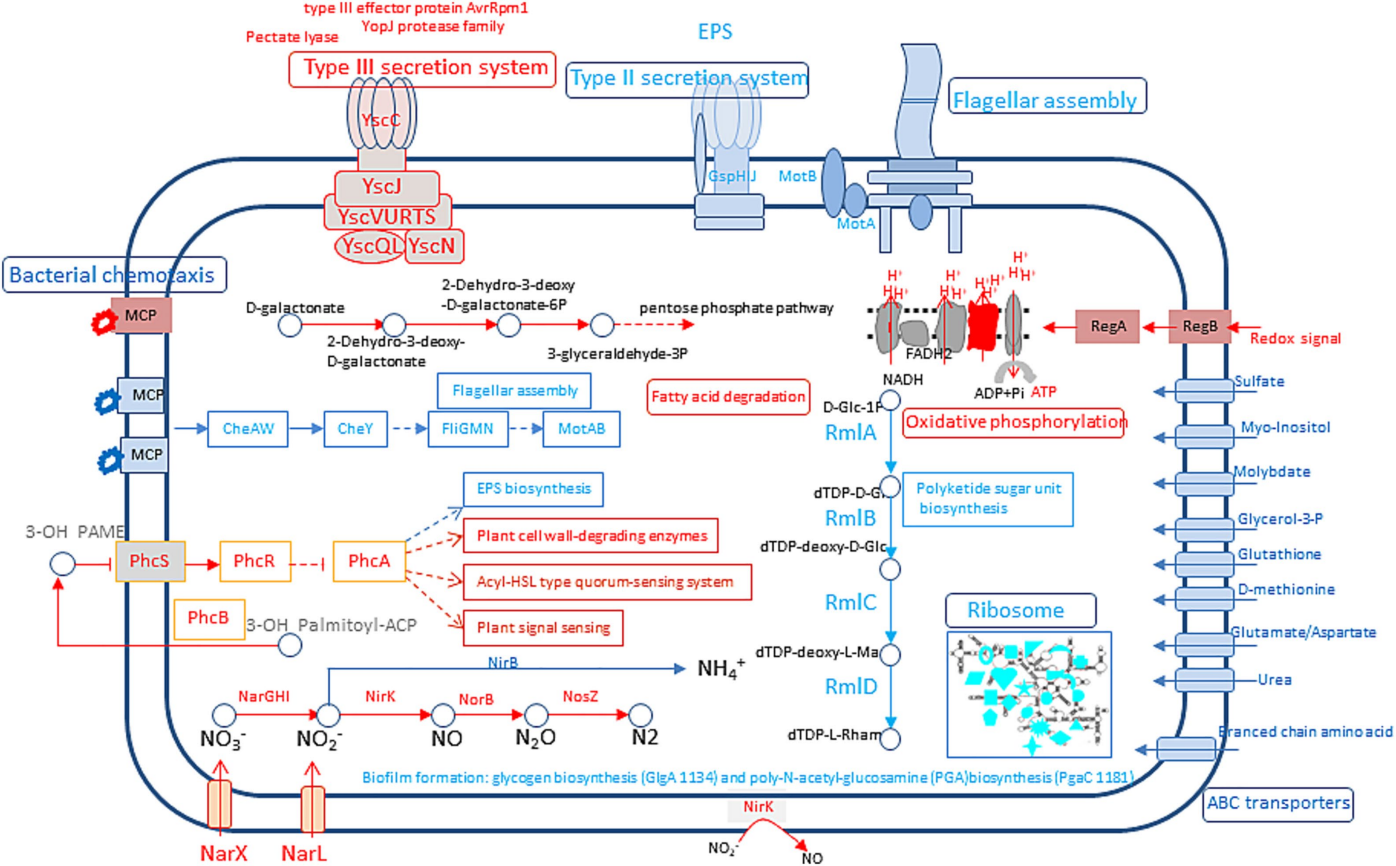
Overview of pathways and functions affected by galactose-containing MGRLS medium compared to MGRLS medium. Functions marked in red are upregulated while those in blue are downregulated.

Extracellular polysaccharide (EPS), a key virulence factor in RSSC, is primarily composed of N-acetyl-galactosamine, with rhamnose-rich polysaccharides and glucans as minor constituents. Rhamnose content has been reported to be approximately eightfold lower than that of N-acetylgalactosamine (Orgambide et al., 1991). Transcriptomic analysis revealed that XpsR, a transcriptional activator of the eps gene cluster, was down-regulated across all monosaccharide treatments. Most EPS biosynthesis genes remained unaltered; however, epsD was significantly down-regulated in galactose, while epsE was up-regulated in xylose (Supplementary Table S5). As for biofilm formation, genes related to glycogen biosynthesis (GlgA gene 1,134, log2FC:-2.15) and poly-N-acetyl-glucosamine (PGA) biosynthesis (PgaC 1,181 log2FC:-1.09) were significantly down-regulated in galactose (Supplementary Table S5; Figure 5). Collectively, these results indicate that none of the monosaccharides tested markedly up-regulated EPS biosynthetic genes.

#### 3.4.2 Almost all of the MCPs sensor and flagellar assembly were down-regulated

Bacteria can detect and respond to a wide range of chemical stimuli, including amino acids, saccharides, and organic acids. Bacterial chemotaxis is also a key precursor to ecological interactions, such as infection and root colonization, during cell motility and signal transduction. Surprisingly, the addition of monosaccharide caused most of the MCPs to be significantly down-regulated, except 928MCP in galactose, 800MCP in the L-arabinose sample (Supplementary Table S6). During the signal transduction of bacterial chemotaxis, we also found that most genes were down-regulated in the main pathway to flagellar assembly, such as gene cheY.

#### 3.4.3 Non-significant upregulation of secondary metabolite biosynthetic genes

Ralfuranones, a class of aryl-furanone secondary metabolites associated with virulence in *Ralstonia solanacearum*, are synthesized from two phenylalanine-derived precursors through the action of the furanone synthase RalA and the aminotransferase RalD. These enzymes catalyze the formation of the common intermediate ralfuranone I in strains such as GMI1000 and OE1-1. Homologs of the key biosynthetic genes ralA (ID 1687) and ralD (ID 1683) were identified in the WXQ_10 genome. Although transcriptome data indicated a discernible upregulation of these genes, their expression levels did not reach statistical significance under any monosaccharide treatment at the sampled time points (Supplementary Table S7).

### 3.5 qPCR analysis of some virulent genes

**Figure 6:**
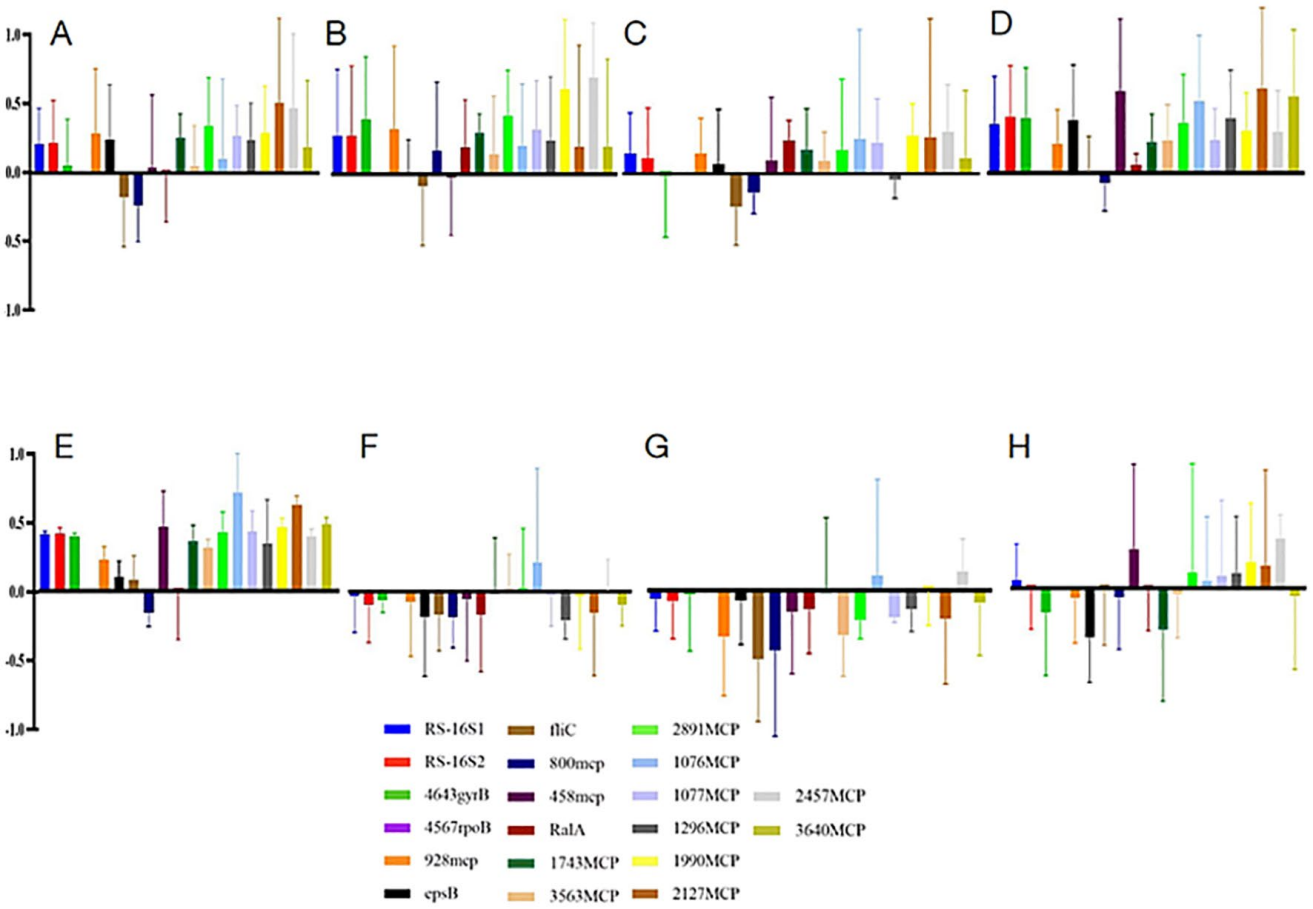
qPCR analysis of strain WXQ_10 cultured with monosaccharide compared with MGRLS medium without monosaccharide. **(A)** Mannose. **(B)** Arabinose. **(C)** Xylose. **(D)** Sucrose control, **(E)** Glucose, **(F)** Galactose, **(G)** Maltose, **(H)** Fructose. The PCR amplified fragment (from left to right): RS-16S1, RS-16S2, 4643gyrB, 4567rpoB, 928mcp, epsB, fliC, 800mcp, 458mcp, ralA, 1743 mcp, 3,563 mcp, 2,891 mcp, 1,076 mcp, 1,077 mcp, 1,296 mcp, 1990 mcp, 2,127 mcp, 2,457 mcp, and 3,640 mcp.

To explore potential links between chemotaxis and virulence regulation, we selected 13 MCP genes and three virulent genes (epsB, fliC, and ralA) to perform quantitative real-time PCR (qPCR) analysis (Supplementary Table S8). The 16S rRNA gene was used as the reference, with expression normalized using two primer pairs targeting distinct regions of the gene. Due to inconsistent results observed in maltose-treated samples, these data were excluded from further analysis. Most of the genes in galactose and fructose samples were down-regulated, different from the above five treatments. About the expression of many mcp genes, 1076mcp and 2457mcp were highly expressed in all samples (Figure 6).

The expression pattern of the epsB gene was similar to that of reference genes. Furthermore, based on the transcriptome sequencing, genes related to type III effectors were significantly up-regulated. EPS might prevent the secretion of effectors, which play important roles in digesting the plant cell walls.

The fliC gene, which encodes flagellin and governs bacterial motility, was down-regulated in all treatments except glucose. Expression analysis in mannose and xylose suggested a potential correlation between fliC and mcp800. In contrast, ralA, involved in the biosynthesis of the phytotoxin ralfuranone, was up-regulated in arabinose and xylose, but not significantly, and down-regulated in the other five monosaccharide treatments; however, the values were nearly zero except for the galactose and maltose samples, and ralA in the galactose and maltose samples was not significantly down-regulated. The expression profile derived from qPCR diverged from the transcriptome data, in which ralA exhibited only modest up-regulation across all samples—a difference which may be due to the different sampling batches and differing time points at which samples were collected. However, comparing the activity of bacterial cells based on the data of the first two internal standards (16S rRNA primers), we found they were also downregulated in galactose and maltose samples (Figures 6F,G).

### 3.6 Plant virulence test by the extract of different monosaccharide-containing fermented media

**Figure 7:**
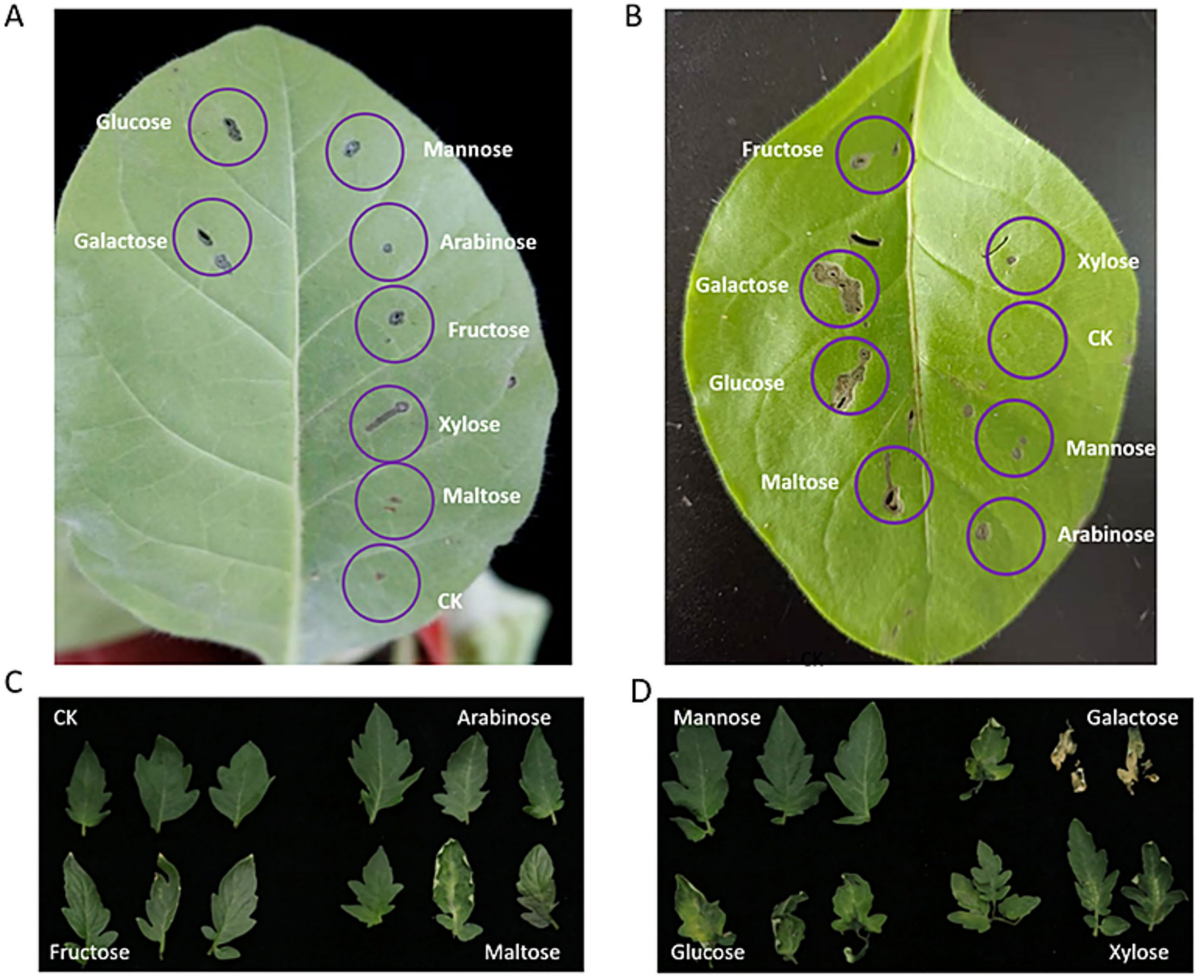
Cell death induced by metabolites cultured in monosaccharides on tobacco leaves and tomato leaves. **(A, B)** In vivo injection of tobacco leaves performed with a sterile syringe; **(C, D)** Detached tomato leaves incubated with petioles submerged in the test solution.

To assess the virulence of secondary metabolites induced by monosaccharides in RSSC, we extracted metabolites from cultures grown in media supplemented with different sugars. These extracts were infiltrated into tobacco leaves or used to immerse detached tomato leaf petioles. The extract derived from galactose-grown cultures exhibited the strongest virulence, causing severe tissue damage and cell death in tobacco leaves (Figures 7A,B). Furthermore, *in vitro* assays using detached tomato leaves confirmed that the galactose-derived extract led to extensive maceration and rotting (Figures 7C,D). Besides galactose, extracts from glucose and maltose cultures also showed measurable phytotoxicity, whereas those from arabinose and mannose did not induce significant symptoms. These findings on the differential effects of monosaccharides are consistent with those previously reported by Ishikawa et al. (2019).

## 4 Discussion

The secretion of allelochemicals from plant roots plays a key role in soil health and soil-borne disease. The goal of this study was to investigate the role of monosaccharides released from tobacco roots on the production of the virulence factors of RSSC. This would enhance our understanding of the ecological effects of plant root exudates in plant-microbe interactions and help to reveal the relationship between tobacco bacterial wilt and allelochemicals that accumulate from root exudates, which will provide new insight into disease control.

Several virulent factors contribute to bacterial wilt disease development. These virulence factors include production of extracellular polysaccharide (EPS), a consortium of plant cell wall-degrading enzymes, twitching and swimming motility, chemotaxis, and several dozen effectors secreted by a type 3 secretion system (T3SS) (Denny, 2006). *R. solanacearum* virulence factors are regulated by a complex interlocking cascade involving quorum sensing and undefined plant signals (Mole et al., 2007; Schell, 2000). Two major virulence factors, EPS and T3SS, are controlled by a global regulator, PhcA, via the quorum-sensing molecule 3-OH-palmitic acid methyl ester (3-OH-PAME) (Flavier et al., 1997; Genin et al., 2005). However, under the culture condition (OD600 = 1.0), the cell concentration was not thick enough to activate the quorum-sensing system. Furthermore, gene3462encoded acyl homoserine lactone synthase, and gene3463 encoded LuxR family transcriptional regulator, quorum-sensing system regulator, were also not significantly up-regulated. The mechanism of causing the higher secretion of effectors by the addition of galactose has not been found.

The expression of specific methyl-accepting chemotaxis proteins (MCPs) was altered at the mRNA level in response to several monosaccharides, indicating a role in chemotactic sensing rather than carbon utilization. Fructose, galactose, glucose, xylose, and maltose up-regulated MCP1076, confirming their role as chemoattractants, and none acted as chemorepellents.

EPS was an unusual exopolysaccharide virulence factor of the phytopathogen RSSC (Huang and Schell, 1995). The promoter alone is responsible for the regulated transcription of an eps operon composed of epsAPBCDE (Huang and Schell, 1995). EPS I, a > 1,000 kDa acidic polymer composed of a repeating trimeric unit of 2-N-acetylgalactosamine, 2-N-acetylgalactos-aminuronic acid, and 2-N-acetyl-2,4,6-trideoxygalactose modified at the N4 position with 3-OH butyric acid (Orgambide et al., 1991). Theoretically, adding galactose should promote the production of EPS. Unexpectedly, the expression of most of the EPS genes was not up-regulated. On the contrary, some genes were down-regulated. EPS is not required for root invasion or growth *in planta* tissue, but is required for wilting and killing, because it blocks water flow in the xylem (Huang et al., 1995; McGarvey et al., 1999). The reason for the down-regulated EPS gene might be that the WXQ_10 cell was existing in the middle stage of cell invasion into the plant cell and the digestion of the cell wall of plant cells.

A consortium of plant cell wall-degrading enzymes also played an important role in the disease process, especially hemicellulose-related genes were significantly upregulated. Plant cell wall-degrading polygalacturonases (PGs) are significant virulence factors for this pathogen. PehA (an endo-PG), or PehB (an exo-PG), or both PehA and PehB are proven to be virulent on wounded eggplants by a deleted mutant (Qi Huang and Allen, 2000). Gene823 (pectate lyase, PL3 family) was significantly up-regulated in galactose (log2FC:1.91), helping to digest the plant cell walls. Besides the digestion of plant cells, some enzymes can be induced to digest the cell wall of themselves or other bacteria. For example, gene 927 (peptidoglycan lyase, GH23) can only be expressed in galactose.

Nitric oxide (NO) and other reactive nitrogen species (RNS) are immensely important signaling molecules in plants, being involved in a range of physiological responses (Durner et al., 1998). In plants, RNS appear to induce genes involved in antioxidative responses as well as those associated with resistance to pathogens (Sarkar et al., 2014). In addition, RNS may contribute to programmed cell death as part of the hypersensitive response to pathogen attack. However, no NOS gene or protein has been found to date in higher plants (Fröhlich and Durner, 2011; Jeandroz et al., 2016). Like the RSSC in solanaceae plants, the endophytes in plants might be the source of RNS. This is why the nitrogen metabolism was active when many effectors were secreted under the induction of galactose.

The coordinated up-regulation of the Type III secretion system (T3SS) alongside the down-regulation of motility (e.g., Methyl-Accepting Chemotaxis Proteins, MCPs) and exopolysaccharide (EPS) biosynthesis represents a pivotal lifestyle shift essential for successful infection in phytopathogens such as *Pseudomonas syringae*, *Ralstonia solanacearum*, and *Xanthomonas* spp. (Monteiro et al., 2012; Planas-Marquès et al., 2020; Serapio-Palacios and Finlay, 2020). This transcriptional reprogramming optimizes resource allocation during host invasion. Specifically, once the pathogen reaches the infection site, suppressing energy-intensive processes like chemotaxis and flagellar motility conserves ATP (Genin and Denny, 2012). Concurrent down-regulation of EPS genes, a metabolically costly pathway, redirects carbon and energy toward the assembly and function of the T3SS (Landry et al., 2020). Moreover, reduced EPS production may facilitate a more aggressive, planktonic-like state that promotes direct contact with host cells, thereby ensuring efficient effector delivery through the T3SS while avoiding physical obstruction by a dense extracellular matrix (Peeters et al., 2013).

## 5 Conclusion

Root exudates, as a key source of organic inputs in the rhizosphere, significantly influence soil health and crop production. *Ralstonia solanacearum* is an important soil-borne plant pathogen responsible for bacterial wilt disease in solanaceous crops. In this study, we examined the transcriptional response of *R. solanacearum* to several root-derived monosaccharides to assess their effect on the expression of virulence-related genes. RNA-seq and qPCR analyses revealed that galactose markedly alters the physiological state of the pathogen, inducing a condition resembling that during plant infection. Compared with several monosaccharides, induction of galactose can significantly up-regulate the expression of type III effectors. Notably, galactose also activated nitrogen metabolism pathways, leading to the production of nitrous oxide and other reactive nitrogen species, which may—like reactive oxygen species—enhance pathogenicity. Furthermore, the metabolites induced by galactose media can cause cell death through injection into the leaf of the tobacco plant.

## Data Availability

The draft whole genome sequence was deposited in NCBI (BioProject PRJNA796546) accession number JAKKIF000000000.

## Footnotes

1
http://lowelab.ucsc.edu/tRNAscan-SE/

2
http://www.vicbioinformatics.com/software.barrnap.shtml

3
http://deweylab.github.io/RSEM/

4
www.majorbio.com

## Supplementary material

SUPPLEMENTARY FIGURE S1 The number of DEGs of mRNA and sRNA and Venn analysis. The number of DEGs of mRNA and sRNA (S1A), mRNA (S1B), sRNA (S1C) in every monosaccharide samples compared to sucrose control; The number of more than 1 transcript (TPM, Transcripts Per Million reads) of mRNA and sRNA (S1D), mRNA (S1E), sRNA (S1F) in seven monosaccharides samples was shown in Venn analysis.

SUPPLEMENTARY FIGURE S2 Volcano of DEGs in samples compared with sucrose samples.

SUPPLEMENTARY FIGURE S3 PCA analysis between samples.
