# Supplementary material for: Root exudate monosaccharides modulate the pathogenicity of Ralstonia pseudosolanacearum
Source: Front Microbiol. 2026 Jan 7;16:1662342. doi: 10.3389/fmicb.2025.1662342 (PMC12819619; doi:10.3389/fmicb.2025.1662342)

## Slide 1
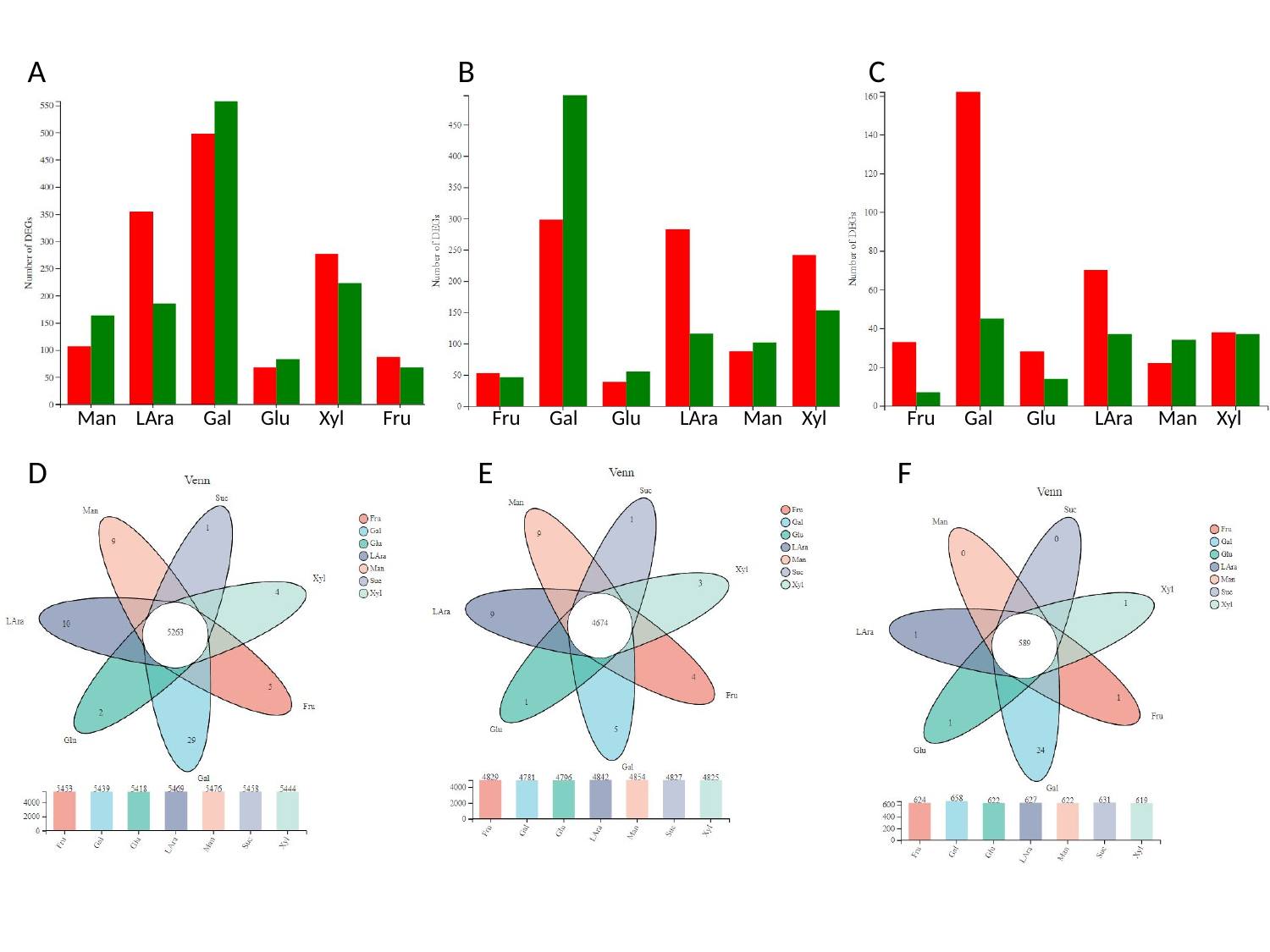

A
B
C
Man LAra Gal Glu Xyl Fru
 Fru Gal Glu LAra Man Xyl
 Fru Gal Glu LAra Man Xyl
D
E
F

## Slide 2
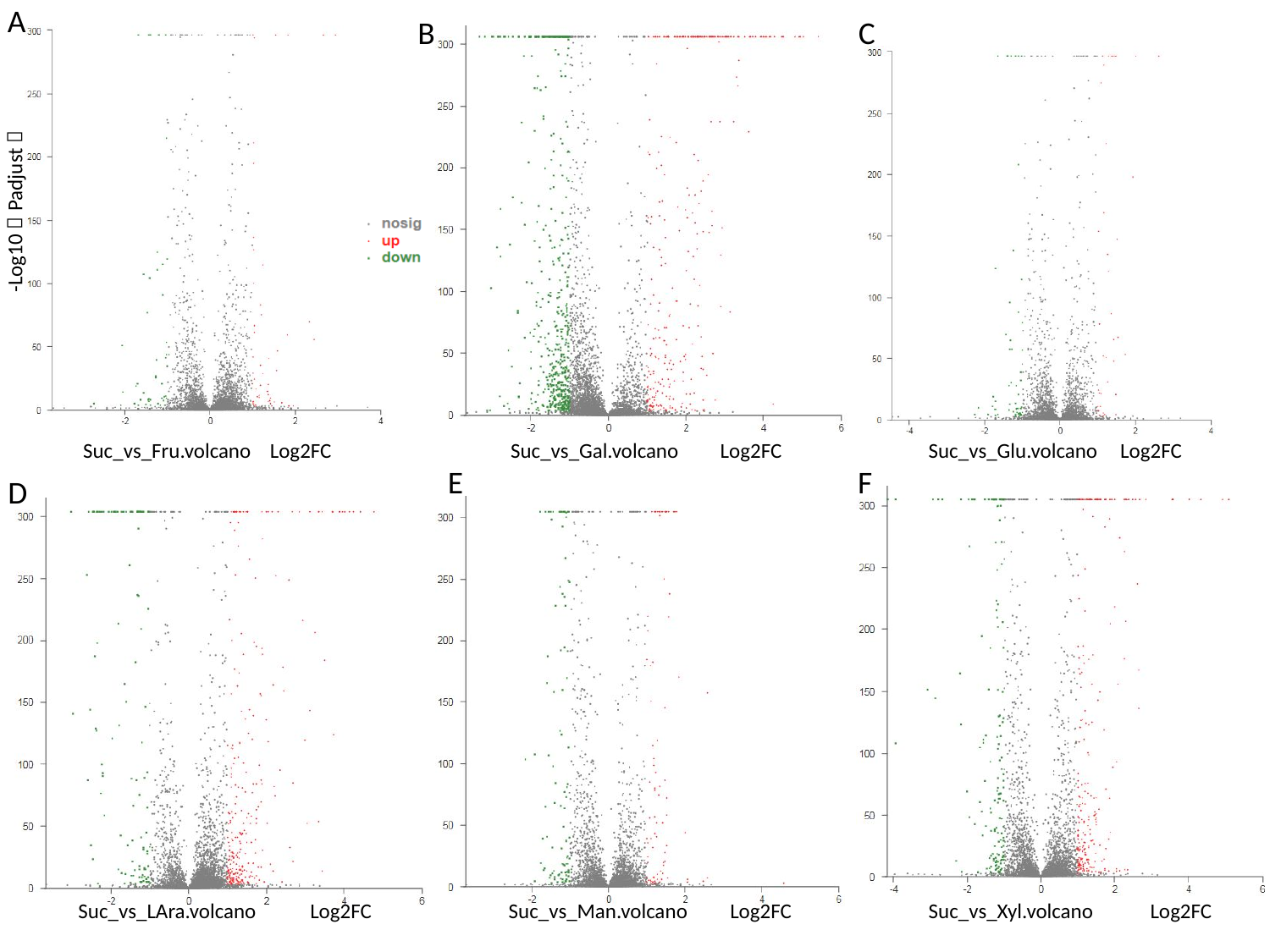

A
B
C
-Log10（Padjust）
Suc_vs_Fru.volcano
Log2FC
Suc_vs_Gal.volcano
Log2FC
Suc_vs_Glu.volcano
Log2FC
E
F
D
Suc_vs_LAra.volcano
Log2FC
Suc_vs_Man.volcano
Log2FC
Suc_vs_Xyl.volcano
Log2FC

## Slide 3
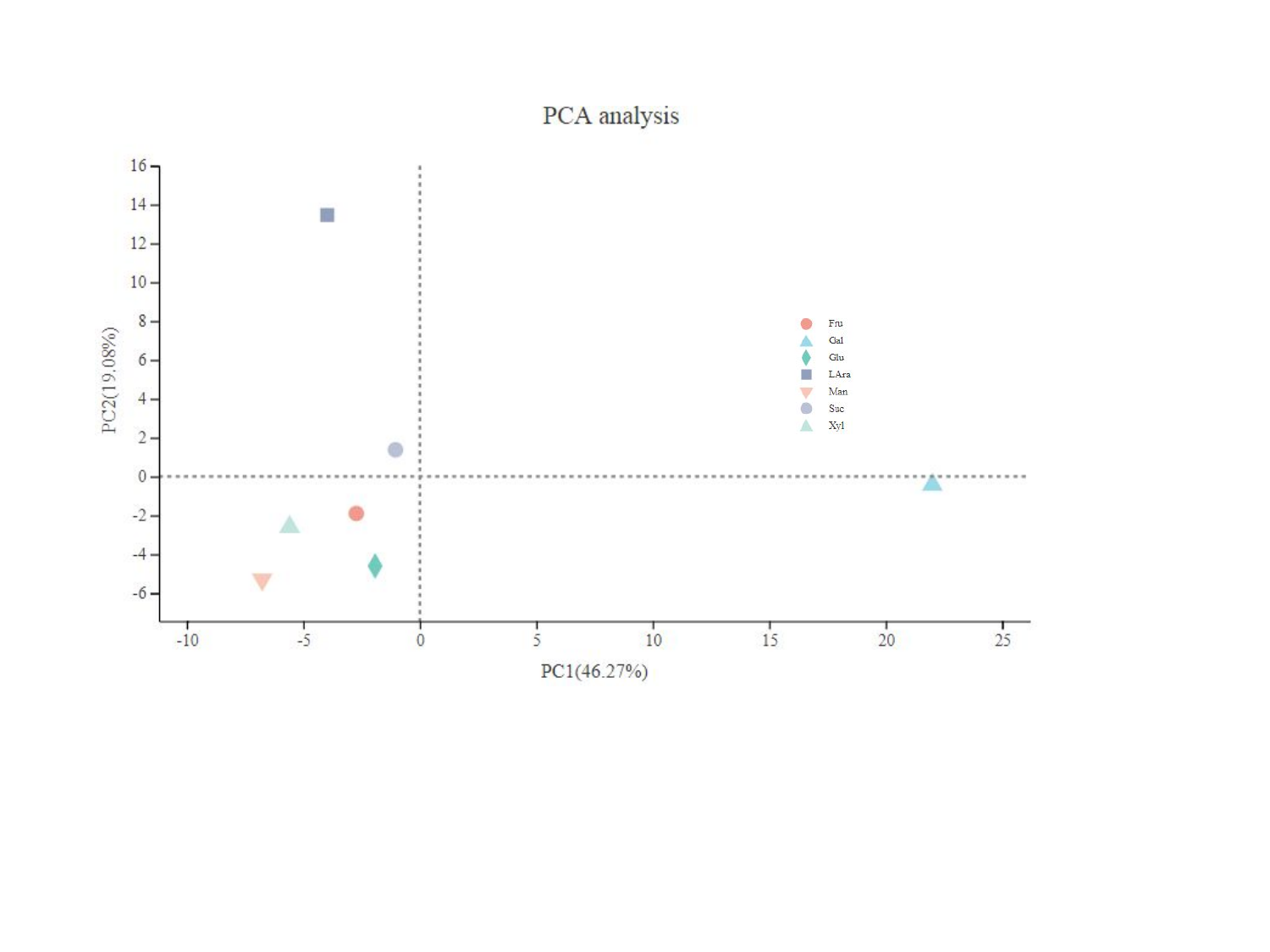

Supplement: SUPPLEMENTARY FIGURE S1 — The number of DEGs of mRNA and sRNA and Venn analysis. The number of DEGs of mRNA and sRNA (S1A), mRNA (S1B), sRNA (S1C) in every monosaccharide samples compared to sucrose control; The number of more than 1 transcript (TPM, Transcripts Per Million reads) of mRNA and sRNA (S1D), mRNA (S1E), sRNA (S1F) in seven monosaccharides samples was shown in Venn analysis. [file Presentation_1.PPTX]
