# Supplementary material for: Root exudate monosaccharides modulate the pathogenicity of Ralstonia pseudosolanacearum
Source: Front Microbiol. 2026 Jan 7;16:1662342. doi: 10.3389/fmicb.2025.1662342 (PMC12819619; doi:10.3389/fmicb.2025.1662342)
Supplement: Supplementary file 2 [file Table_1.docx]

**Supplementary materials**

Table S1 Genome assembly of strain WXQ_10

Table S2 data statistics of non-coding RNA prediction

Table S3 quality control data of the transcriptome

Table S4. log2FC of genes related with plant cell wall degrading

Table S5. log2FC of genes related with EPS biosynthesis

Table S6. log2FC of MCPs and genes related with flagellar assembly

Table S7. log2FC genes involved in the biosynthesis of ralfuranone

Table S8. Primers used in this study for qPCR

Table S1 Genome assembly data statistics

| property | contig | scaffold |
| --- | --- | --- |
| total sequence number | 209 | 207 |
| total sequence length | 5,638,120 | 5,638,120 |
| Max sequence length | 250,432 | 250,432 |
| Min sequence length | 506 | 506 |
| N20 | 150,957 | 153,228 |
| N50 | 91,683 | 94,037 |
| N90 | 19,653 | 19,653 |
| N number | 0 | 189 |
| N rate | 0 | 0 |
| GC content % | 67.05 | 67.05 |
| sequences greater than 1 kb | 169 | 167 |

Table S2 data statistics of non-coding RNA prediction

| RNA_Type | Copy Number | Average_length  (bp) | Total_length  (bp) | percent of genome(%) |
| --- | --- | --- | --- | --- |
| 5S rRNA | 1 | 108 | 108 | 0.0019 |
| 16S rRNA | 1 | 1532 | 1532 | 0.0272 |
| 23S rRNA | 1 | 2876 | 2876 | 0.0510 |
| tRNA | 51 | 78 | 4002 | 0.0710 |
| ncRNA | 82 | 136 | 11185 | 0.1984 |

Table S3 quality control data of the transcriptome

|  | Raw data | | | | | Clean data | | | | |
| --- | --- | --- | --- | --- | --- | --- | --- | --- | --- | --- |
| Sample Name | reads (million) | Bases (Mbp) | Error Rate (%) | Q20 (%) | Q30 (%) | Reads  (million) | Bases (Mbp) | Error Rate (%) | Q20(%) | Q30(%) |
| Fru | 35 | 5293 | 0.016 | 95.76 | 90.7 | 34 | 4135 | 0.0128 | 98.19 | 94.46 |
| Gal | 33 | 5030 | 0.0163 | 95.43 | 90.42 | 32 | 3749 | 0.0125 | 98.33 | 94.81 |
| Glu | 35 | 5332 | 0.0159 | 95.83 | 90.83 | 34 | 4198 | 0.0127 | 98.22 | 94.51 |
| LAra | 31 | 4661 | 0.0159 | 95.83 | 90.8 | 30 | 3631 | 0.0127 | 98.21 | 94.5 |
| Man | 35 | 5234 | 0.0159 | 95.88 | 90.78 | 34 | 4155 | 0.0129 | 98.14 | 94.31 |
| Suc | 33 | 5001 | 0.016 | 95.75 | 90.71 | 32 | 3911 | 0.0128 | 98.18 | 94.45 |
| Xyl | 35 | 5307 | 0.0157 | 96.0 | 90.97 | 34 | 4260 | 0.0129 | 98.13 | 94.31 |
| Average | 33 | 5122 | 0.016 | 95.78 | 90.74 | 32 | 4005 | 0.013 | 98.2 | 94.48 |

Table S4. log_2_FC of genes related with plant cell wall degrading

| Family | gene | Xyl | Gal | Man | Fru | Glu | LAra |
| --- | --- | --- | --- | --- | --- | --- | --- |
| GT5 | 1134 | no | -2.15 | 1.78 | no | no | no |
| GH23 | 1823 | no | -2.03 | no | no | no | no |
| GH109 | 1968 | no | -2.02 | no | no | no | no |
| GT20 | 1996 | no | -1.78 | 1.09 | no | no | no |
| GT2 | 1800 | no | -1.57 | no | no | no | no |
| CE1 | 2506 | 1.21 | -1.52 | 1.41 | no | no | no |
| GT2 | 2354 | no | -1.48 | no | no | no | no |
| GT30 | 1807 | no | -1.36 | no | no | no | no |
| GT104 | 3624 | -1.02 | -1.25 | -1.1 | no | no | no |
| GH13_16 | 1132 | no | -1.18 | no | no | no | no |
| CBM63 | 3184 | no | -1.12 | no | no | no | no |
| CE10 | 2898 | no | -1.11 | no | no | no | no |
| GT2 | 1181 | no | -1.09 | no | no | no | no |
| AA6 | 2236 | no | -1.05 | no | no | no | no |
| GH103 | 1352 | no | -1.05 | no | no | no | no |
| GH13_3 | 1133 | no | -1.04 | 1.35 | no | no | no |
| CE7 | 4126 | no | -1.04 | no | no | no | no |
| GT4 | 1276 | no | -1 | no | no | no | no |
| AA1_3 | 2375 | no | 1.07 | no | no | no | 1.03 |
| GH109 | 4645 | no | 1.15 | no | no | no | no |
| CE3 | 2699 | no | 1.16 | no | no | no | no |
| CE4 | 571 | no | 1.59 | no | no | no | no |
| PL3_1 | 823 | no | 1.9 | no | no | no | no |
| GH23 | 927 | no | 2.4 | no | no | no | no |
| AA1_3 | 3914 | no | 2.42 | no | no | no | no |
| GT20 | 1586 | no | 2.44 | no | no | no | no |
| PL6 | 1736 | no | 3.07 | no | no | no | -1.44 |
| GH23 | 4518 | no | no | no | no | no | -1.42 |
| GT32 | 3345 | no | no | no | no | no | 1.01 |
| GH109 | 249 | no | no | no | no | no | 1.08 |
| GH32 | 2660 | no | no | no | no | no | 1.1 |
| AA3_2 | 4436 | no | no | no | no | no | 1.11 |
| GH28 | 3211 | 1.03 | no | no | no | no | 1.12 |
| GH73 | 3383 | no | no | no | no | no | 1.25 |
| AA1_3 | 3942 | no | no | no | no | no | 1.44 |
| GH109 | 247 | 1.31 | no | no | no | no | 1.52 |
| CE10 | 1678 | no | no | 1.23 | 1.7 | 1.5 | 1.56 |
| CE8 | 4318 | no | no | no | no | no | 1.76 |
| GH13_9 | 1131 | no | no | 1.16 | no | no | no |
| CE4 | 1180 | no | no | 1.3 | no | no | no |
| CE10 | 2002 | no | no | no | no | -1.07 | no |
| GH28 | 887 | no | no | no | 1.02 | no | no |
| CBM32 | 3029 | 1.31 | no | no | no | no | no |
| CE1 | 1452 | no | yes | no | no | no | no |

CBM: Carbohydrate-Binding Modules; CE: Carbohydrate Esterases; GH: Glycoside Hydrolases; AA: Auxiliary Activities; GT: Glycosyl Transferases; PL: Polysaccharide Lyases. no: not significantly regulated

Table S5. log_2_FC of genes related with EPS biosynthesis

| gene | Gene name | Gal | LAra | Xyl | Man | Fru | Glu |
| --- | --- | --- | --- | --- | --- | --- | --- |
| 1270 | xpsR | -1.16 | -0.43 | -0.69 | -1.18 | -0.84 | -0.56 |
| 1281 | epsF | 0.68 | -0.11 | -0.41 | -0.69 | 0.38 | -0.19 |
| 1282 | epsE | -0.62 | 0.39 | 1.08 | 0.46 | 0.58 | 0.60 |
| 1283 | epsD | -1.36 | 0.49 | 0.48 | 0.06 | 0.25 | -0.02 |
| 1284 | epsC | -0.22 | 0.37 | -0.28 | -0.05 | 0.00 | -0.05 |
| 1285 | epsB | 0.01 | 0.41 | 0.32 | 0.28 | 0.45 | 0.31 |
| 1286 | epsP | 0.66 | 0.75 | 0.91 | -0.49 | 0.91 | 0.10 |
| 1287 | epsA | -0.74 | 0.46 | 0.52 | -0.06 | 0.68 | 0.22 |

Table S6. log_2_FC of MCPs and genes related with flagellar assembly

| gene |  | Xyl | Gal | Man | Fru | Glu | Ara |
| --- | --- | --- | --- | --- | --- | --- | --- |
| 1076 | AKZ26812.1 | no | no | no | no | no | no |
| 1077 | WP_071012720.1 | -1.25 | -1.79 | no | no | no | -1.15 |
| 1192 | WP_019719190.1 | -1.13 | -1.03 | -1.13 | no | no | -1.14 |
| 1296 | WP_011004317.1 | no | no | no | no | no | no |
| 1743 | ESS50341.1 | -1.19 | no | -1.67 | -1.56 | -1.69 | -1.19 |
| 1990 | WP_020829916.1 | no | no | no | no | no | no |
| 2127 | CUV30795.1 | no | no | no | no | no | no |
| 2457 | WP_020830342.1 | no | no | no | no | no | no |
| 2891 | CUV57823.1 | no | no | -1.11 | no | no | no |
| 3563 | WP_016722673.1 | no | no | no | -1.03 | -1.27 | no |
| 3640 | WP_043876687.1 | no | no | no | no | no | no |
| 458 | WP_019718844.1 | no | no | no | no | no | no |
| 800 | AKZ25549.1 | no | no | no | no | no | 2.41 |
| 928 | WP_028854319.1 | no | 1.01 | no | no | no | no |
| 1695MotB | WP_011004660.1 | -1.25 | -1.51 | -1.05 | -1.13 | no | no |
| 1696CheY | WP_011004659.1 | -1.19 | -1.52 | -1.21 | no | -1.32 | no |
| 1700CheR | WP_011004655.1 | no | -1.99 | -1.5 | -2.04 | -1.35 | -1.01 |
| 1701CheD | WP_011004654.1 | -1.46 | -1.56 | -1.38 | -1.06 | -2.14 | no |

no: not significantly regulated

Table S7. log_2_FC genes involved in the biosynthesis of ralfuranone

| Sample name | Log2FC ralA1687 | Log2FC ralD1683 |
| --- | --- | --- |
| Galactose | 0.26 | 0.11 |
| Glucose | -0.05 | 0.62 |
| Fructose | 0.19 | 0.72 |
| L-Arabinose | 0.24 | 0.77 |
| Mannose | 0.24 | 0.22 |
| Xylose | 0.45 | 0.72 |

Table S8. Primers used in this study for qPCR

| Primer Name | Seqence(5'-3') |
| --- | --- |
| 16S-RS10-F1 | TGGTAGTCCACGCCCTAAACGA |
| 16S-RS10-R1 | AACCCAACATCTCACGACACGA |
| 16S-RS10-F2 | TGTAGCAGTGAAATGCGTAGAG |
| 16S-RS10-R2 | TGTCAAGGGTAGGTAAGGTTTT |
| 4643gyrB-F | ACAGCAGAAACCGCAATCCACA |
| 4643gyrB-R | AGACCATTCACGCACGACACG |
| 4567rpoB-F | CGGGCACGAACTCCATCAGC |
| 4567rpoB-R | CGAGCACGACAAGGGCAAGA |
| 928mcp-F | CTTGGCGTGGCATTCGGCTTTG |
| 928mcp-R | ACAGGTCCTGGCCGGATTGGGT |
| epsB-FW | ATGGTCGAGCTGATGGATA |
| epsB-RV2 | TGGAGCTGCTTGATCGTCTC |
| fliC-FW2 | CAAACGCAAGGTATTCAGAACG |
| fliC-RV2 | ATTGGAAGGTCGTCGAAGCCAC |
| 800mcp-F | CGAGTCGGCGGAGGTCTTGATA |
| 800mcp-R | ATTCAAAGACCCGACCGGCAAA |
| 458 mcp-F | GACGGCATTGCGTTCCAGACCA |
| 458 mcp-R | GGCGGATGGAGGTGACGATGTT |
| ralA-F | TGCGTGCTTGGTGGCGTGATTC |
| ralA-R | GTCAGAACGACCGCTTTGGAGT |
| 1743MCP-F | GACGCTGGCACCGTAGGCTGAA |
| 1743MCP-R | GGCTGGACGACAACAACACGAA |
| 3563MCP-F | GCCGATGCGTCCAACTCCACCA |
| 3563MCP-R | GTGCCTGACCGTCTTCCTGCTC |
| 2891MCP-F | GTTGCTCGCCAGTTTCGGTTTG |
| 2891MCP-R | GCCCAGTTGTCTCGCCTTGTCC |
| 1076MCP-F | CTGCTGCGTCACCTGCTCCATC |
| 1076MCP-R | CGGCATCGCCTTCCAGACCAAT |
| 1077MCP-F | TTGTCGGAACGGGTGATGGTGA |
| 1077MCP-R | CGCTGACGCTGATGTCGGTGTT |
| 1296MCP-F | CGCACGCAGCAGATACCACGAC |
| 1296MCP-R | GCATCACGAGCGACAAGGAACG |
| 1990MCP-F | TGCTTGATGTGGCTGATGGTGC |
| 1990MCP-R | CCTGCTGATGACGCTCGTGGTG |
| 2127MCP-F | GCGTTCCAGACCAACATCCTCG |
| 2127MCP-R | GATGCCGCTGCTCTGCTCTTCC |
| 2457MCP-F | GGCATCAACGGCAGCAGCAAGA |
| 2457MCP-R | CGCTTGACCGCCACCACCACTT |
| 3640MCP-F | GGTCTGGAACGCAATGCTGTCG |
| 3640MCP-R | GGTGGTCGGCAACGATGAGGTG |
